# Identification of key influence factors and an empirical formula for spring snowmelt-runoff: A case study in mid-temperate zone of northeast China

**DOI:** 10.1038/s41598-018-35282-x

**Published:** 2018-11-16

**Authors:** Lin Tian, Hongyan Li, Fengping Li, Xiubin Li, Xinqiang Du, Xueyan Ye

**Affiliations:** 1 Key Laboratory of Groundwater Resources and Environment, Ministry of Education, Jilin University, Changchun, 130021 China; 2 Baishan Power Plant of State Grid Xin Yuan Company Limited, Huadian, 132400 China

## Abstract

Because of the unique climate characteristics, the runoff law in mid-temperate zone is very different from other regions in spring. Accurate runoff simulation and forecasting is of great importance to spring flood control and efficient use of water resources. Baishan reservoir is located in the upper Second Songhua River Basin in Northeast China, where snowmelt is an important source of runoff that contributes to the water supply. This study utilized long-term hydrometeorological data, in the contributing area of Bashan reservoir, to investigate factors and time-lag effects on spring snowmelt and to establish a snowmelt-runoff model. Daily precipitation, temperature, and wind data were collected from three meteorological stations in this region from 1987–2016. Daily runoff into the Baishan reservoir was selected for the same period. The snowmelt period was identified from March 23 to May 4 through baseflow segmentation with the Eckhardt recursive digital filtering method combined with statistical analyses. A global sensitivity analysis, based on the back propagation neural network method, was used to identify daily radiation, wind speed, mean temperature, and precipitation as the main factors affecting snowmelt runoff. Daily radiation, precipitation, and mean temperature factors had a two-day lag effect. Based on these factors, an empirical snowmelt runoff model was established by genetic algorithm (GAS) to estimate the snowmelt runoff in this area. The model showed an acceptable performance with coefficient of determination (R^2^) of 73.6%, relative error (Re) of 25.10%, and Nash-Sutcliffe efficiency coefficient (NSE) of 66.2% in the calibration period of 1987–2010, while reasonable performance with R^2^ of 62.3%, Re of 27.2%, and NSE of 46.0% was also achieved during the 2011–2016 validation period.

## Introduction

Snow is an important form of precipitation in the surface water cycle because snow accumulation and ablation processes occur over much global land area near the latitude of 40°N, especially in the inland areas that account for roughly one-sixth of the world’s population and about one quarter of the global Gross Domestic Product^1^. The mean annual snowfall supplying streamflow in China is 3.45 × 10^11^ m^3^ and the mean nnual winter snow water storage is found to be 5.36 × 10^10^ m^3^ or about 3.5 cm in snow depth^2^. Runoff from glaciers (glacier runoff) has been an active area of research in recent years^3,4^; however, runoff from snow (snowmelt) is different from glacier runoff. Glaciers play a multi-year regulatory role for runoff, while snowmelt plays a seasonal adjustment role^5^. There is a large amount of seasonal snow cover in the mid-temperate zone of northeast China, due to its unique climate characteristics. Low reflectivity snow may accumulate, which allows for a couple months of seasonal snow cover and a delayed runoff effect compare to normal rainfall^6^. Hence, there are often two peaks in the annual runoff duration curve of a seasonal permafrost basin. One occurs during the spring snowmelt period, while the other occurs in the summer flood season. It is known that global climate change is having substantial effects on hydrologic systems, accompanied with an increasing risk of floods caused by rapid snowmelt. In contrast, spring precipitation may hard to satisfy irrigation and water demand for the farming and sowing season. Hence, accurate streamflow simulation and forecasting is of great importance to spring flood control and water resources availability for irrigation, reservoir operation, and hydropower generation in spring, especially in mid-temperate zone of northeast China.

Generally, snowmelt runoff is the result of many heat transfer processes to the snowpack which from the falling snow deposition^7,8^. The snowpack melting process has seasonal and periodic characteristics that are influenced by natural factors including temperature and radiation, as well as underlying surface factors such as vegetation types/covers^9^. The snowmelt runoff usually shows the diurnal cycle of a peak and a valley, which lag behind the cycles of meteorological factors. The length of the lag time depends on the size of the watershed area and the conditions of the underlying surface of the region. It is very complex to clarify the mechanism of snowmelt runoff and its spatial and temporal variations, especially in areas where gauged data are not available. Snowmelt runoff in cold regions has been simulated using several models distributed snowmelt models^10–14^, and models based on mass and energy balance such as the degree-day Snowmelt Runoff Model (SRM)^15–17^, Snow Thermal Model (SNTHERM)^18,19^. The SRM model is simple and easy to access but requires remote sensing data as input, while distributed snowmelt models are impractical to use in data-sparse regions. Empirical models have relatively limited data requirements and are simple to operate, but can achieve the required accuracy in many cases^20,21^.

The present study takes Baishan basin, the main watershed of the mid-temperate zone in northeast China, as an example aimed to (1) identify the impact factors of spring snowmelt runoff and their time lag effects on runoff, and (2) establish an empirical snowmelt runoff model to estimate the spring snowmelt runoff. The primary goal of this study was to provide an effective tool for snowmelt runoff simulation, which could be used to examine reasonable water resources operations in mid-temperate zone of northeast China.

## Study Area

### Location

**Figure 1:**
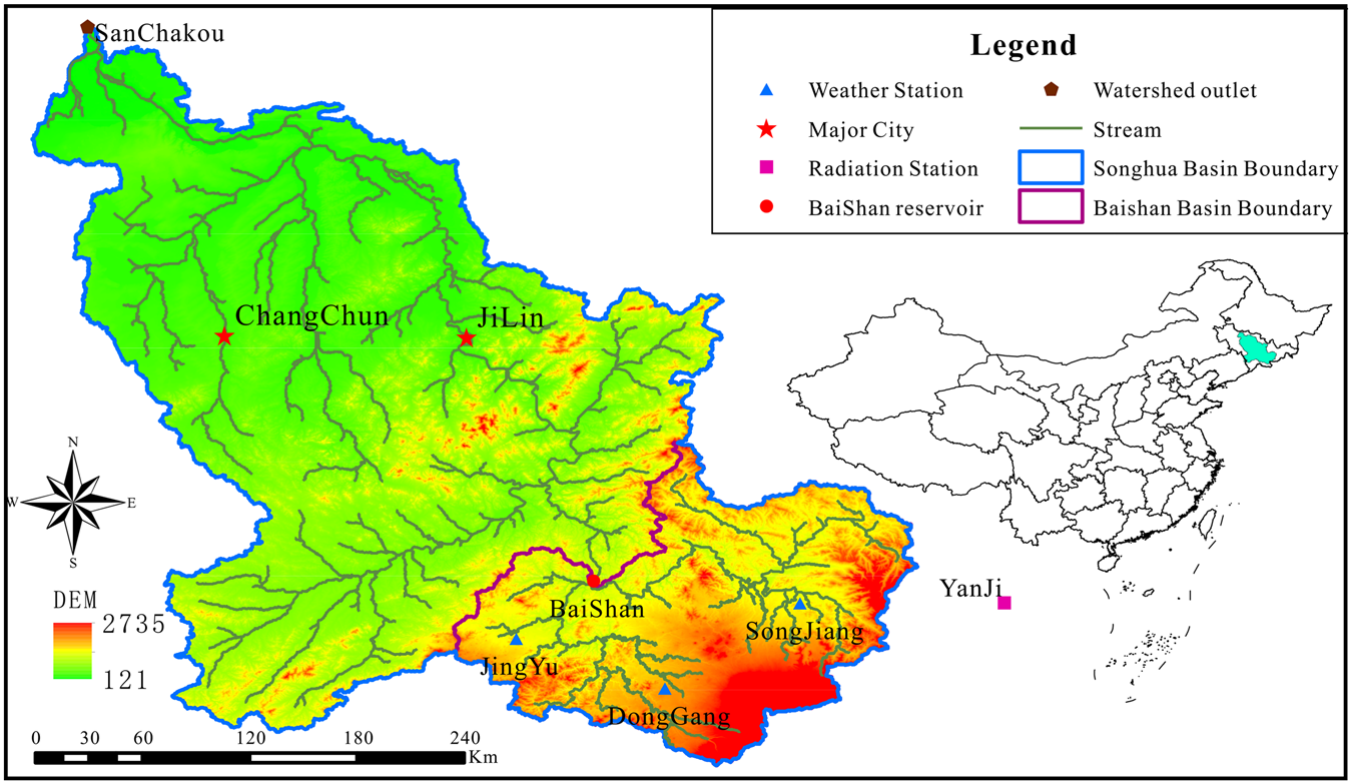
Location of the study area (above Baishan reservoir).

The drainage area contributing runoff to the Baishan reservoir is selected as the study area (Fig. 1). Baishan reservoir is located at the junction of two counties (i.e. Huadian and Jingyu) in the eastern mountain area of Jilin Province and on the main stem of the Second Songhua River, northeastern China. The Baishan watershed has a drainage area of 18,724 km^2^ with flow originating from Changbai Mountain and travels 255.7 km to the reservoir. A hydropower project was started on October 1958, but suspended on June 1962. In 1975, the government proposed to resume it, and finally completed on June 1992. Baishan hydropower Station is the first developed hydropower station on the main stream of the Second Songhua River, which accounts for 25.9% of the area of the Second Songhua River basin, with an annual runoff of 75.3 × 10^8^ m^3^ and a storage capacity of 65.1 × 10^8^ m^3^.

The study area is located in the Changbai mountain area and is surrounded by mountains with dense forests and a narrow valley. The study area is within the continental monsoon climate zone with long and cold winters, humid and rainy summers, and distinct seasons. The annual average temperature of the study area is 4.3 °C, and maximum annual average freezing time is up to 135 days. Therefore, this area belongs to a typical seasonal frozen soil area.

### Selection of snowmelt factors and data sources

The snowmelt runoff process is different from ordinary runoff processes because the main factor to consider for the runoff process is rainfall, but the snowmelt runoff process is affected by many factors^22^. Temperature is generally considered to be a good index of the heat transfer processes associated with melting of snowpack^23^. Therefore, a temperature approach is often used to estimate the snowmelt based on the energy-balance method^24,25^. However, many scholars^26–28^ believe that both temperature and precipitation affect snowpack. It has also been found that snow properties such as snow depth, snow density and the grain size in some snowmelt models are most sensitive to solar radiation energy flux and incoming longwave radiation^29,30^. The US Army Corps of Engineers proposed the HEC-1(HEC-HMS) snowmelt runoff model based on a regression analysis method, the process of water cycle is divided into four aspects: precipitation loss, direct runoff, basic flow and river channel confluence, which includes temperature, radiation, wind speed, and other factors to calculate snowmelt. Because of this background, we selected radiation, wind speed, precipitation, minimum temperature, mean temperature, and maximum temperature as parameters in the empirical formula that are adjusted to simulate snowmelt runoff. These factors are generally the readily available data even in regions where data are scarce.

Daily runoff data, of good consistency and integrity, are collected for the Baishan reservoir Station from the Baishan Power Plant of State Grid. Meteorological data (from 1987–2016) are derived from National Meteorological Information Center, China Meteorological Administration. Wind speed, precipitation and temperature data are from three climate stations (i.e. Jingyu, Donggang, and Songjiang) shown in Fig. 1. Thiessen polygons were defined around the three sites so that the data could be spatially weighted. The result of segmentation is generally reasonable according to elevation and river system at Jingyu, Donggang, and Songjiang with the corresponding weight coefficients being 0.21, 0.31, and 0.48, respectively, used for meteorological data processing. Radiation data from Yanji station, shown in Fig. 1, were used in the empirical model.

## Methodology

Sensitivity analysis quantitatively investigates the extent of changes in dependent variables caused by independent variables. It has been an important research tool when studying the response of hydrological factors to climate change (IPCC 1995). In particular, the Artificial Neural Networks method (ANN) is one of the most widely used methods^31–34^. In the present study, the global sensitivity analysis method based on back propagation (BP) neural network^35^ is used to verify the relationships between factors affecting snowmelt runoff, analyze the time-lag effect of each factor, and distinguish the critical factors. On this basis, the empirical formula of snowmelt runoff is optimized by genetic algorithms method (GAS) to estimate the snowmelt runoff of the study area.

### Global sensitivity analysis method based on BP network

ANNs are effective for simulating many complex nonlinear relationships. ANNs have been widely used in hydrology, and have been verified as reliable in research^36,37^. A BP network is a multi-layer feedforward training method based on error back propagation. It is also one of the most widely used artificial neural network algorithms because of its clear physical concept and ease of operation. Design optimization of the BP algorithm, parameter selection, precision evaluation and other details, are described in detail by Yuan *et al*.^38^.

This study adopts the global sensitivity analysis method using a BP network, and studies the effect of each variable on the dependent variable with consideration of the interactions between different variables. The zeroing disturbance of an independent variable is introduced to deduce the change of a dependent variable, so that we can define the sensitivity of the dependent variable to the independent variable and conduct global sensitivity analysis based on multiple factors coupling. Global sensitivity analysis considers the interaction between the variables and therefore is more suitable for most of the practical problems^39,40^.

Supposed that there is random variable Y and a series of independent variables ${{\rm{X}}}_{1},{{\rm{X}}}_{2},\ldots ,{{\rm{X}}}_{i},\ldots ,{{\rm{X}}}_{n}$, they are all on the basis of a time series in a system. Assuming a nonlinear deterministic mapping ${\rm{Y}}=f({{\rm{X}}}_{1},{{\rm{X}}}_{2},\ldots ,{{\rm{X}}}_{i},\ldots ,{{\rm{X}}}_{n})$, and the sample is expressed as follows:

1 $$\begin{array}{rcl}{y}_{1} & = & f({X}_{1,1},{X}_{2,1},\ldots ,{X}_{i,1},\ldots ,{X}_{n,1})\\ {y}_{2} & = & f({X}_{1,2},{X}_{2,2},\ldots ,{X}_{i,2},\ldots ,{X}_{n,2})\\ \ldots  &  & \\ {y}_{k} & = & f({X}_{1,k},{X}_{2,k},\cdots ,{X}_{i,k},\ldots ,{X}_{n,k})\\ \ldots  &  & \\ {y}_{m} & = & f({X}_{1,m},{X}_{2,m},\ldots ,{X}_{i,m},\ldots ,{X}_{n,m})\end{array}\}$$

where m is sample size and n is dimension of independent variables.

if ${X}_{i,k+1}={X}_{i,k}+{\rm{\Delta }}{X}_{i,k\to k+1}$; ${y}_{k+1}={y}_{k}+{\rm{\Delta }}{y}_{k\to k+1}$, formula (1) can be expressed as:

2 $$\begin{array}{ll}{y}_{k+1} & =\,{y}_{k}+\Delta {y}_{k\to k+1}\\  & =\,f({x}_{1,k}+\Delta {x}_{1,k\to k+1},{x}_{2,k}+\Delta {x}_{2,k\to k+1},\ldots ,{x}_{i,k}\\  & \,\,\,+\,{\rm{\Delta }}{x}_{i,k\to k+1},\ldots ,{x}_{n,k}+{\rm{\Delta }}{x}_{n,k\to k+1})\end{array}$$

According to Taylor’s mean value theorem, the Taylor expansion of multivariate function is carried out for the right side of formula (2), and the second-order partial derivation is preserved, and the formula (3) is obtained as:

3 $$\begin{array}{c}f({x}_{1,k}+{\rm{\Delta }}{x}_{1,k\to k+1},{x}_{2,k}+{\rm{\Delta }}{x}_{2,k\to k+1},\ldots ,{x}_{i,k}+{\rm{\Delta }}{x}_{i,k\to k+1},\ldots ,{x}_{n,k}+{\rm{\Delta }}{x}_{n,k\to k+1})\\ \,\approx \,f({x}_{1,k},{x}_{2,k},\ldots ,{x}_{i,k},\ldots ,{x}_{n,k})+({\rm{\Delta }}{X}_{1,k\to k+1}\frac{\partial }{\partial {X}_{1}}+{\rm{\Delta }}{X}_{2,k\to k+1}\frac{\partial }{\partial {X}_{2}}+\ldots \\ \,\,\,+\,{\rm{\Delta }}{X}_{i,k\to k+1}\frac{\partial }{\partial {X}_{i}}+\ldots +{\rm{\Delta }}{X}_{n,k\to k+1}\frac{\partial }{\partial {X}_{n}})|{}_{({X}_{1,k},{X}_{2.k},\ldots ,{X}_{i,k},\ldots ,{X}_{n,k})}+\frac{1}{2!}({\rm{\Delta }}{X}_{1,k\to k+1}\frac{\partial }{\partial {X}_{1}}\\ \,\,{+\Delta {X}_{2,k\to k+1}\frac{\partial }{\partial {X}_{2}}+\ldots +{\rm{\Delta }}{X}_{i,k\to k+1}\frac{\partial }{\partial {x}_{i}}+\ldots +{\rm{\Delta }}{X}_{n,k\to k+1}\frac{\partial }{\partial {X}_{n}})}^{2}|{}_{({X}_{1,k},{X}_{2,k},\mathrm{...},{X}_{i,k},\ldots ,{X}_{n,k})}\end{array}$$

Combined with the formula (2) and formula (3), we can get formula (4):

4 $$\begin{array}{ccc}{\rm{\Delta }}{y}_{k\to k+1} & = & ({\rm{\Delta }}{X}_{1,k\to k+1}\frac{\partial }{\partial {X}_{1}}+{\rm{\Delta }}{X}_{2,k\to k+1}\frac{\partial }{\partial {X}_{2}}+\ldots +{\rm{\Delta }}{X}_{i,k\to k+1}\frac{\partial }{\partial {X}_{i}}+\ldots \\  &  & +\,{\rm{\Delta }}{X}_{n,k\to k+1}\frac{\partial }{\partial {X}_{n}})|{}_{({X}_{1,k},{X}_{2,k},\ldots ,{X}_{i,k},\ldots ,{X}_{n,k})}+\frac{1}{2!}({\rm{\Delta }}{X}_{1,k\to k+1}\frac{\partial }{\partial {X}_{1}}+{\rm{\Delta }}{X}_{2,k\to k+1}\frac{\partial }{\partial {X}_{2}}+\ldots \\  &  & {+{\rm{\Delta }}{X}_{i,k\to k+1}\frac{\partial }{\partial {X}_{i}}+\ldots +{\rm{\Delta }}{X}_{n,k\to k+1}\frac{\partial }{\partial {X}_{n}})}^{2}|{}_{({X}_{1,k},{X}_{2,k},\ldots ,{X}_{i,k},\ldots ,{X}_{n,k})}\end{array}$$

When $\frac{{\Delta }{y}_{k\to k+{1}}}{{y}_{k}}={\eta }_{k}$, then ${\eta }_{k}$ is the mapping of the independent variable ${x}_{i,k}$ and its incremental ${\rm{\Delta }}{x}_{i,k\to k+1}$, can be expressed as:

5 $${\eta }_{k}=g({x}_{1,k},{x}_{2,k},\ldots ,{x}_{i,k},\ldots ,{x}_{n,k},{\rm{\Delta }}{x}_{1,k\to k+1},{\rm{\Delta }}{x}_{2,k\to k+1},\ldots ,{\rm{\Delta }}{x}_{i,k\to k+1},\ldots ,{\rm{\Delta }}{x}_{n,k\to k+1})$$

For $g(\,\ast \,)$ this kind of non-linear mapping relationship, as long as the sample size *m* is large enough, the BP network can be used for identification. If the BP network has completed the mapping relationship $g(\,\ast \,)$ recognition through training, introduced the zeroing disturbance ${\rm{\Delta }}{x}_{i,k\to k+1}=0$. This is used to calculate the influence of dependent variables without considering the increment of independent variables, expressed as:

6 $$\begin{array}{c}{\eta ^{\prime} }_{i,k}=(g({x}_{1,k},{x}_{2,k},\ldots ,{x}_{i,k},\ldots ,{x}_{n,k},\Delta {x}_{1,k\to k+1},\Delta {x}_{2,k\to k+1},\ldots \Delta {x}_{i1,k\to k+1},\\ \,\,\,\,\,0,\Delta {x}_{i+1,k\to k+1}\ldots ,\Delta {x}_{n,k\to k+1})\end{array}$$

The resulting disturbance of dependent variables is: ${\beta }_{i,k}={\eta }_{i,k}-{\eta ^{\prime} }_{i,k}$, the overall disturbance effect of all samples is sensitivity:

7 $${\beta }_{i}=\frac{{\sum }_{k=1}^{m}|{\beta }_{i,k}|}{m}$$

According to this sensitivity, we can identify the delay effect and the main sensitive factor that affect the snowmelt factor.

### GAS

GAS is an efficient global search method to solve the problem by simulating the natural evolution process. It can automatically acquire and accumulate knowledge about the search space in the search process, and adaptively control the search process to find the optimal solution^41,42^. The basic idea of GAS is to start the search from a set of random initial solutions, namely the “population”. A population consists of a certain number of individuals encoded by “gene”. Each individual in the population is called a “chromosome”. After the first generation of a population, each subsequent generation produce better according to the principle of survival of the fittest and evolution. Chromosomes in each generation, by means of crossover and mutation, produces new chromosome, known as “offspring”. After several generations, the algorithm converges on the best chromosomes. It is very likely that the optimal individual in the last population after decoding is the optimal solution or the near optimal solution of the problem.

GAS has the advantages of wide applicability and global optimization, and it is a natural algorithm constructed by simulating the biological world. It takes probability selection as the main means, and does not involve complex mathematical knowledge or inner rules of the problem itself. Therefore, GAS can deal with any complicated target function and constraint condition. GAS uses probability search rather than path search, so it is a global search in the sense of probability. Therefore, the optimal solution can be obtained in theory and avoid falling into local minima.

Combined optimization is one of the most basic and important research and application fields of GAS. The combinatorial optimization is to find a solution which satisfies the given constraint conditions and makes the value of the objective function value maximum or minimum in discrete and finite mathematical structures. In general, combinatorial optimization problems usually have a large number of local extreme points, which are often non-differentiable, discontinuous, multi-dimensional, constrained, and highly nonlinear complex problems. As a new type of stochastic search and optimization method for simulating biological evolution, GAS has been widely applied in combinatorial optimization field^43,44^. The GAS parameter optimization method of the empirical formula of snowmelt runoff is essentially the problem of using GAS to optimize the combination. The purpose of using GAS to estimate the parameter is to find the optimal value of the combined parameter so as to minimize the error (E) between the output value of the model and the expected value of the sample, namely:

8 $$E=\frac{{1}}{{2}}\sum _{k={1}}^{K}\sum _{i={1}}^{I}{({y}_{i,k}-{d}_{i,k})}^{{2}}$$

where *K* is sample size; *I* is dimension of independent variables; *y* is the output value; *d* is the expected output value.

When *I* = 1, the formula (8) is simplified to:

9 $$E=\frac{{1}}{{2}}\sum _{k={1}}^{K}{({y}_{i,k}-{d}_{i,k})}^{{2}}$$

The parameters of GAS can be adjusted in the process of determining the parameters of the empirical formula in order to make choice of the optimal results.

## Results and Discussion

### Amount of accumulated snow

Snow has some unique physical properties that can affect the energy exchange between the ground and atmosphere and the radiation balance. For instance, the high reflectivity of snow can improve the surface reflectance by 30–50%, while the low thermal conductivity reduces the surface sensible heat transfer into the air. During the snowmelt period, the high latent heat of snow increases the absorption of the surface energy and subsequently reduces the ground surface temperature.

**Figure 2:**
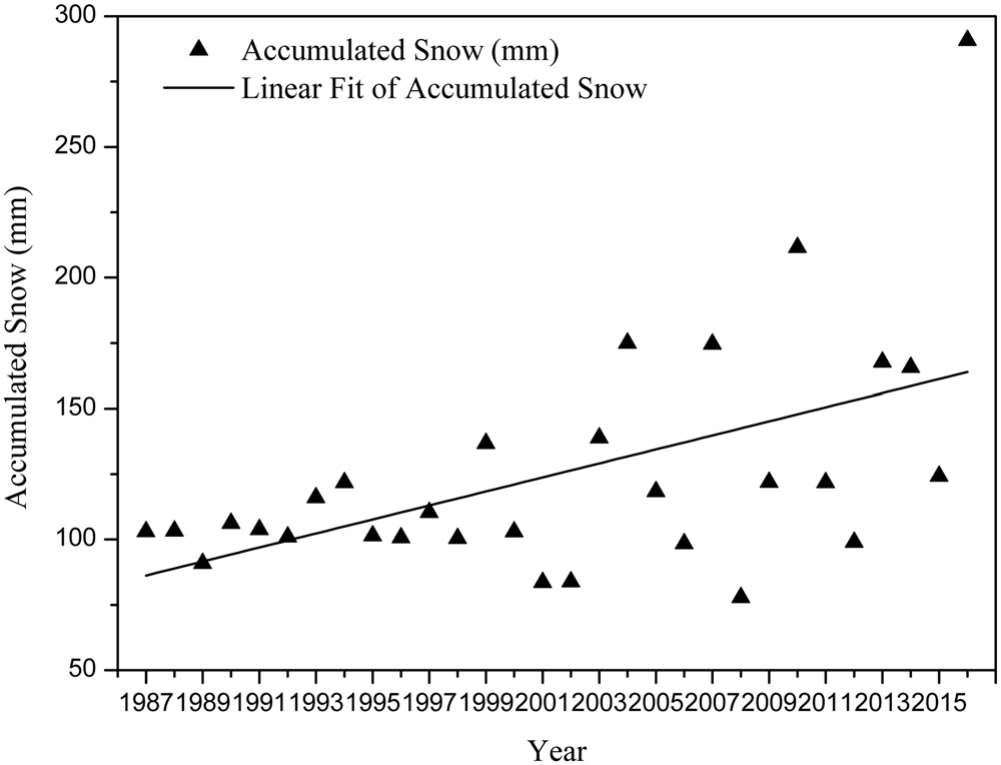
Interannual variation of accumulated snow from 1987 to 2016.

The study area is located in the seasonal frozen soil region, so the cumulative effect of accumulated snow has a great influence on snowmelt runoff. The quantity of snowmelt is also dependent upon the condition of the snowpack itself. If there is a small amount of accumulated snow in winter, the response of snowmelt runoff in summer might not be obvious. Therefore, we analyzed the interannual variability of accumulated snow in the study area before carrying out other work. We calculated the monthly precipitation from November 1 in the previous year to March 31 in the current year as the accumulated snow for each year. Figure 2 illustrates the changes of accumulated snow from 1987–2016, with a mean square deviation of 44.3. It can be found that snow accumulation generally exhibited an increasing trend and have obvious trend of fluctuations, which means accumulated snow plays a more important role in the calculation of snowmelt runoff. There is not any year when the amount of accumulated snow is extremely small, so all the years from 1987–2016 are included in the scope of our study.

### Selection of snowmelt period

In seasonal frozen soil regions, groundwater is also the main source of river runoff. River runoff has two main parts, surface runoff and underground runoff. Surface runoff mainly comes from rainfall and snowmelt in spring. While underground runoff, which is also called baseflow, is mainly dependent on the precipitation and also affected by the geological structure of the underlying surface, the direction of the impervious layer and the water supply of the basin^45^. There are numerous base flow segmentation methods, such as graphic methods^46^, nonlinear storage process^47^, and Eckhardt recursive digital filtering (ECK) method^48^, which is one of the most widely used methods in recent years.

The ECK method is applied for base flow separation to define an appropriate snowmelt period. The equation of the ECK is:

10 $${q}_{t}=\frac{({1}-{I}_{GF,{\max }})\ast a\ast {q}_{t-1}+({1}-a)\ast {I}_{GF,{\max }}\ast {Q}_{t}}{{1}-a\ast {I}_{GF,{\max }}}$$

where *a* is the parameter of filter and *I*_*GF*_, _*max*_ is the maximum groundwater flow index.

Daily runoff data of Baishan reservoir Station from 1971 to 2016 were selected for base flow segmentation, and *I*_*GF*_, _*max*_ was determined to be 0.744 using the minimum sliding method^49^. We conducted a comprehensive analysis on the base flow ratio (i.e. ratio of base flow to total runoff) from March 1 to May 31 for each year of 1987–2016 to identify the snowmelt period.

**Figure 3:**
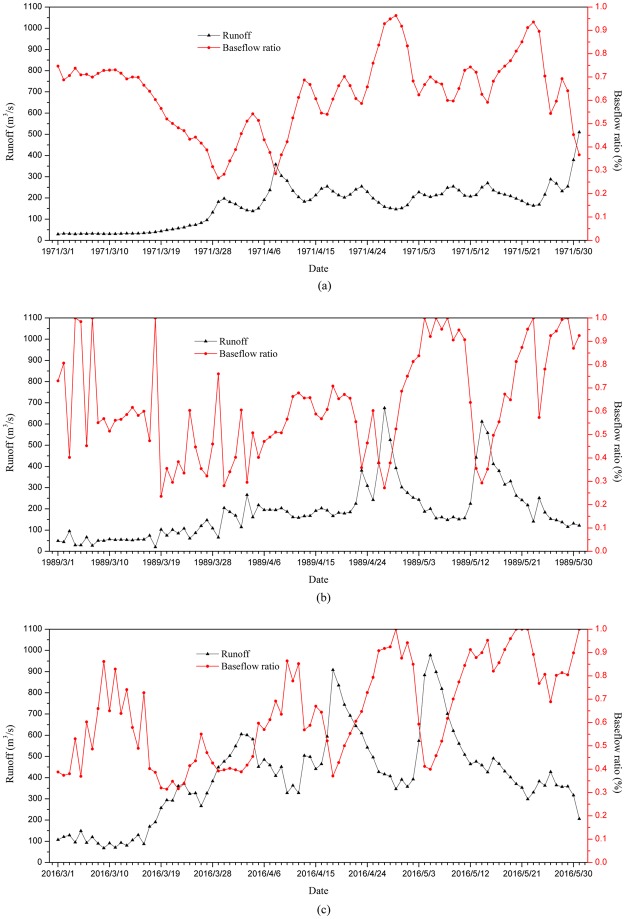
Variation of spring base flow ratio in 1971 (**a**), 1989 (**b**) and 2016 (**c**).

**Figure 4:**
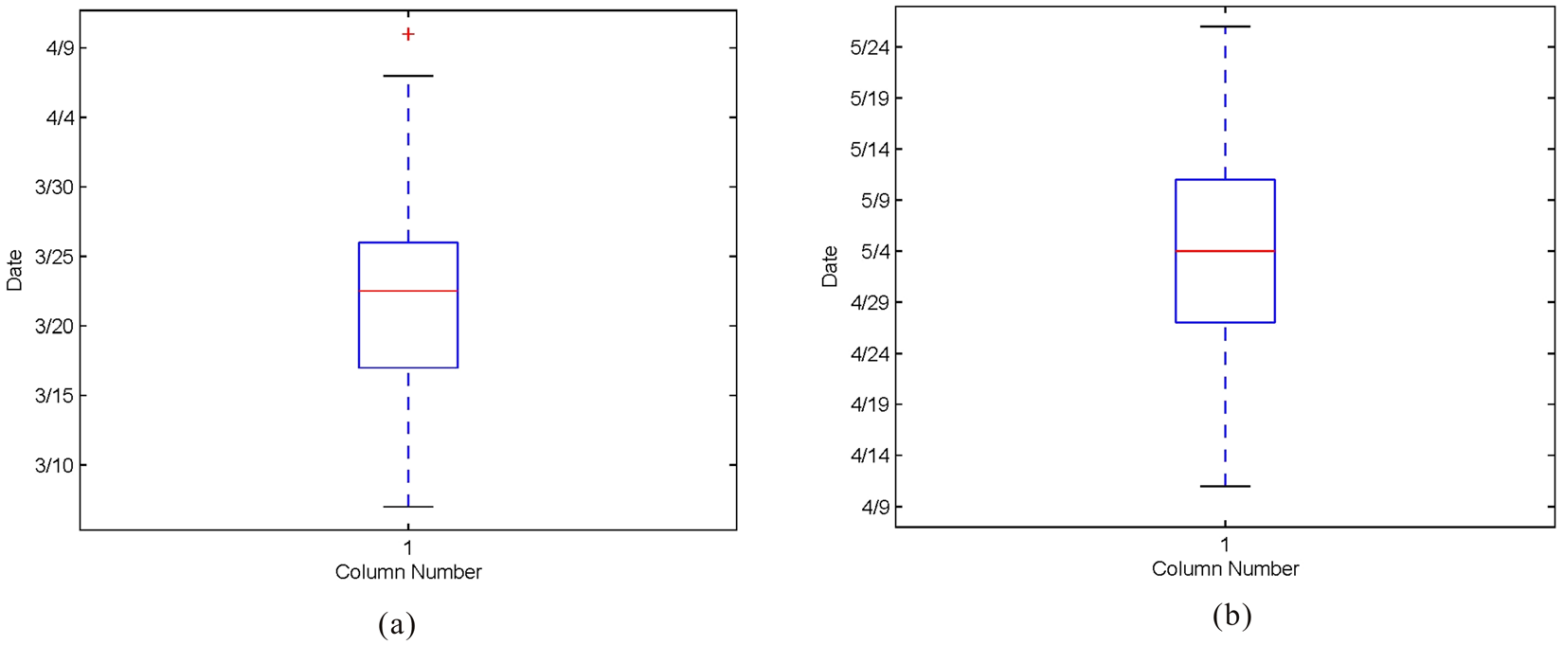
Distributions of snowmelt start dates (**a**) and end dates (**b**) from 1971–2016.

Figure 3 shows 1971, 1989, and 2016 as examples to illustrate that the base flow ratio was significantly lower in late March than the average, while there is an obvious peak from the end of April to early May. In other words, the proportion of surface runoff to total runoff at the end of March to April is higher than for other months. This indicates that the frozen soil can prevent the infiltration of snowmelt before the soil is completely thawed^50^, so that the snowmelt water forms a rapid slope flow and reduces the proportion of the base flow in total runoff. After the frozen soil has thawed, the rapid slope flow above the frozen soil gradually disappears. Consequently, we observed a sharp increase in base flow ratio from the end of April to early May. Therefore, we selected the day in late March when the base flow ratio was lowest as the start date of the snowmelt period for each year, and selected the day from the end of April to May with peak base flow ratio as the end date of snowmelt period. Accordingly, the snowmelt periods were obtained for each year. Based on the distribution of start dates and end dates of snowmelt periods (Fig. 4), we finally selected the median of the numbers from March 23 to May 5 of each year as the snowmelt period to identify the impact factors in the snowmelt process. We determined that the date of snow melting was just around the Spring Equinox, the Spring Equinox sun shines directly on the equator. After that, the direct point of the sun continues to move northward, the day is long and the night is short, and the temperature begins to rise rapidly.

### Model Construction and Result Analysis

#### Sample organization

Measured parameters include daily runoff into Baishan reservoir, total radiation exposure, daily mean wind speed, daily precipitation, daily minimum temperature, daily mean temperature, and daily maximum temperature. Six samples were established to evaluate the time-lag effect of each meteorological factor on runoff. Detailed sample information is listed in Table 1 for solar radiation. The samples for other meteorological factors were organized in the same way.

**Table 1:** Sample information when identifying the time-lag effect of radiation on runoff.

No.	Net input/factors	Net output
1	Solar radiation on the *i-5*th day (SR_T5_)	Runoff (on the *i*th day)
2	Solar radiation on the *i-4*th day (SR_T4_)	
3	Solar radiation on the *i-3*th day (SR_T3_)	
4	Solar radiation on the *i-2*th day (SR_T2_)	
5	Solar radiation on the *i-1*th day (SR_T1_)	
6	Solar radiation on the *i*th day (SR_T0_)	

Subsequently, samples of meteorological factors with time-lags (Table 2) were organized based on the prior global sensitivity analysis, and used to identify factors affecting snowmelt runoff. The impact factors were used to optimize the parameters of the empirical formula with the GAS method.

**Table 2:** Sample information for identifying impact factors (The value of * is obtained from the results of the previous six samples).

No.	Net input/factors(considering time lag effects)	Net output
1	Solar radiation * days ago (SR_T*_)	Runoff (on the day)
2	Wind speed * days ago (Win_T*_)	
3	Precipitation * days ago (Pre_T*_)	
4	Minimum temperature * days ago (LT_T*_)	
5	Mean temperature * days ago (MT_T*_)	
6	Maximum temperature * days ago (HT_T*_)	

#### Parameter selection

Network training was based on an improved BP algorithm that combines the optimized initial weight of GAS^37^ with the limited supervised adjustment of learning rates^51^. According to multiple trials, the hidden layer node is determined as 30–20. The transfer function is an S-type logsig function, and the topology structure is 6-50-30-1with a sample size of 1290. The network initial weight was randomly generated in the [−1, +1] interval. Because the input for the samples have different physical quantities, the dimensions and the orders of magnitude differ. Therefore, all the input of the samples were transformed to a variation range of 0.10 (minimum) and 0.90 (maximum). The related parameters are shown in Table 3.

**Table 3:** Main parameters for BP algorithm training and GAS.

GAS parameters				BP parameters			
Parameter	Value	Parameter	Value	Parameter	Value	Parameter	Value
Population quantity	12 000	Aberration rate	0.05	Maximum of normalization	0.90	Regulating coefficient of learning rate	0.80
Selectance	0.05	Initial area	[−1, 1]	Minimum of normalization	0.10	Initial learning rate	0.01
Crossing-over rate	0.10	Evolution algebra	30	Momentum coefficient	0.80	Average error of network output	0.02

Based on the foregoing parameters, the training times were estimated by trial and error. The algorithm for estimating training times includes (1) when the sample error shows decreases, then add the training times; and (2) when the sample error increases, stop adding the training times so as to avoid excessive training. After repeating trials, when the average output error is <0.02, the network simulation and testing accuracy is regarded as most reasonable. The network training allows the error estimation formula to be:

11 $$\varepsilon  < {(\bar{{\rm{\Delta }}})}^{2}\cdot N$$

where *N* is sample size.

#### Sensitivity analysis

**Figure 5:**
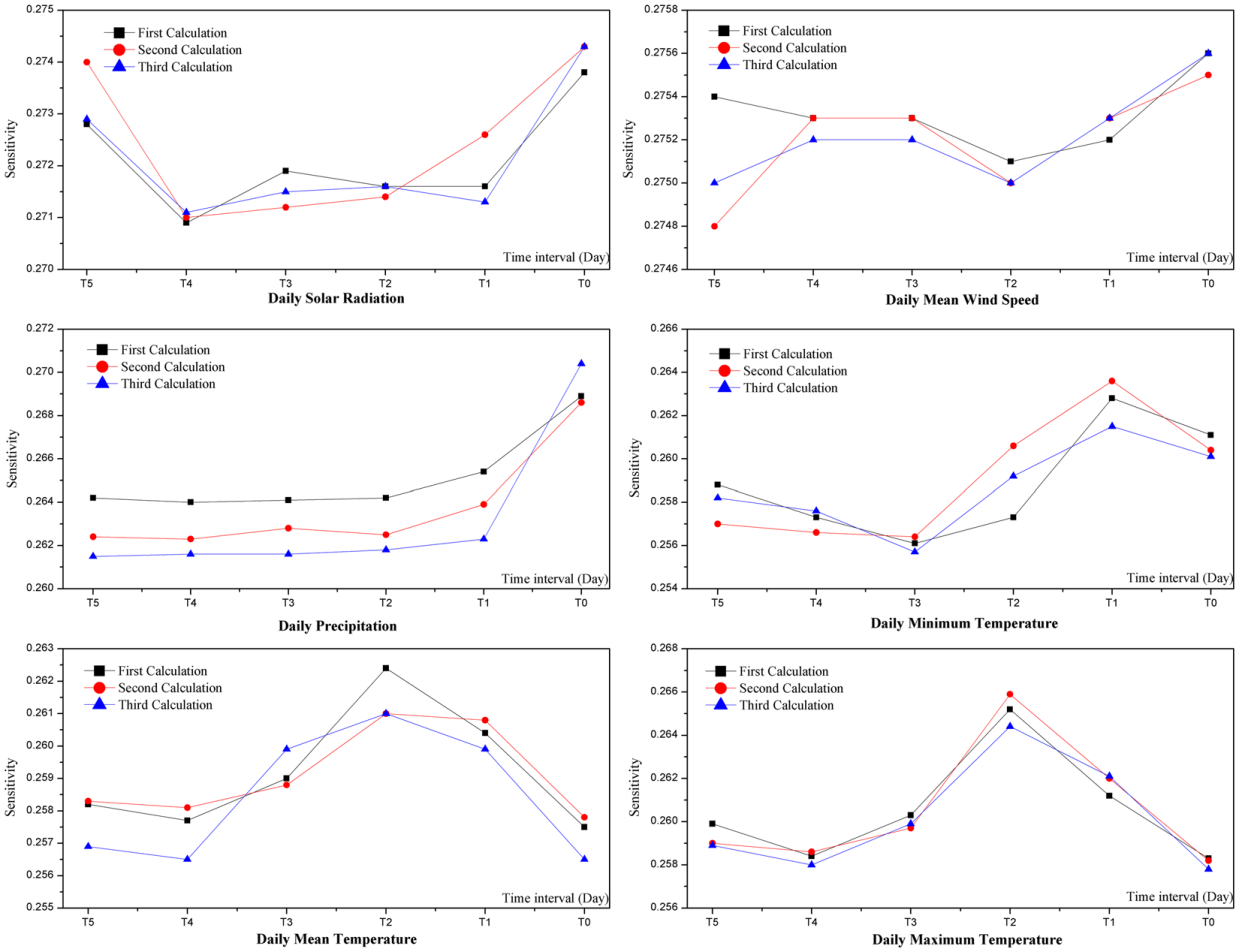
Lag time of each meteorological factor (T0 represent on the day relative to the runoff day; T1 represent yesterday relative to the runoff day, and so on).

First, time-lag effects of each meteorological factor on runoff were analyzed through global sensitivity analysis using the samples as shown in Table 1. Each sample was calculated three times with results of the three calculations shown in Fig. 5. Similarly, the lag periods of each meteorological factor on snowmelt runoff are shown in Table 4.

**Table 4:** Lag time of each meteorological factor on snowmelt runoff.

Parameter	Lag period (day)	Parameter	Lag period (day)
Daily total amount of radiation exposure convergence	0	Daily minimum temperature	1
Daily mean wind speed	0	Daily mean temperature	2
Daily precipitation	0	Daily maximum temperature	2

Different calculations of a single meteorological factor showed similar trends (Fig. 5). The most sensitive factors of snowmelt runoff on the *i*th day selected from each sample were daily total radiation exposure on the *i*th day (SR_T0_), the daily mean wind speed on the *i*th day (W_T0_), the daily precipitation on the *i*th day (P_T0_), the daily minimum temperature on the *i-1*th day (LT_T1_), the daily mean temperature on the *i-2*th day (MT_T2_), and the maximum temperature on the *i-2*th day (HT_T2_).

**Figure 6:**
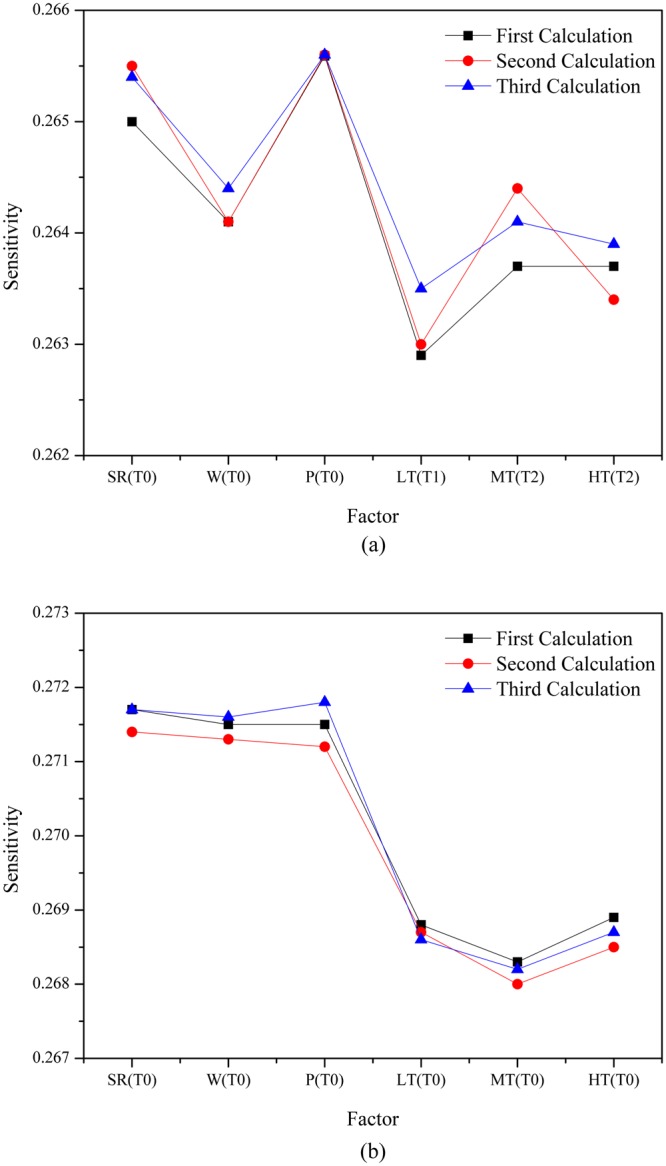
Sensitivity of snowmelt runoff to each meteorological factor considering time-lag effect (**a**) and regardless of time-lag effect (**b**).

Afterwards, SR_T0_, W_T0_, P_T0_, LT_T1_, MT_T2_, and HT_T2_ were selected as the new input for the network through the sample created in Table 2. The results of the three calculations are shown in Fig. 6(a). Snowmelt runoff was more sensitive to SR_T0_ and P_T0_ than to others, but the sensitivities to LT_T1_and HT_T2_ were minimal.

Another sample was organized to verify whether time-lag effects analysis are necessary for each factor. In this sample, SR_T0_, W_T0_, P_T0_, LT_T0_, MT_T0_, and HT_T0_ were selected as the new input of the network and the output of runoff remained the same. Similarly, the results of three calculations were shown in Fig. 6(b), which shows that the sensitivity of the snowmelt runoff to the three temperature factors substantially decreased compared to other factors, which suggests that the time lag recognition of each factor is of great importance.

### Empirical formula fitting

The results of the sensitivity analysis show that snowmelt runoff is more sensitive to SR_T0_, W_T0_, P_T0_, and MT_T2_. Therefore, these factors were used as the independent variables, combined with the amount of accumulated snow (AS), when fitting the empirical formula. The fitted empirical formula would be used to simulate the snowmelt runoff.

A generalized empirical formula for glacier runoff^5^ is as follows:

if $${P}_{t}\ge R{D}_{t},{P^{\prime} }_{t}=R{D}_{t}$$;

if ${P}_{t} < R{D}_{t},{P^{\prime} }_{t}={P}_{t}+({P}_{t-1}-{P^{\prime} }_{t-1})+({P}_{t-2}-{P^{\prime} }_{t-2})+\mathrm{...}+({P}_{t-n}-{P^{\prime} }_{t-n})\le R{D}_{t}$, trial method should be used in determine the value *n* and *P*′;

12 $$R{D}_{t}={x}_{1}{(AS\ast {{x}_{2}}^{i})}^{{x}_{3}}\ast S{R}_{t}^{{x}_{4}}\ast {{{\rm{W}}}_{{\rm{t}}}}^{{{\rm{x}}}_{5}}\ast {\rm{M}}{{T}_{t-2}}^{{x}_{6}}+{x}_{7}{P^{\prime} }_{t}$$

where *t* is time series, from 0 to 42 (from March 23 to May 4), and have 30 cycles from 1987–2016.

***RD*** is the runoff (0.1 mm);

***AS*** is the amount of accumulated snow (0.1 mm), from November 1 of the previous year to March 31;

***i*** is the attenuation index, rounding from 0 to 42 in turn;

***SR*** is the daily total radiation exposure (0.01 MJ/m^2^);

***W*** is the daily wind speed (0.1 m/s);

***MT*** is the daily mean temperature (°F);

***P***′ is the treated daily precipitation (0.1 mm),

***x1***, ***x2***, ***x3***, ***x4***, ***x5***, ***x6*** and ***x7*** is the parameters need to be estimated and optimized with GAS.

The parameters of the formula were first estimated by the data from 1987–2010, and then tested with the data from 2011–2016 to ensure the accuracy of the empirical formula.

The empirical formula with optimized parameters is given as follows:

13 $$R{D}_{t}={0.0004}{(AS\ast {0.968}{{8}}^{i})}^{{0.2918}}\ast S{R}_{t}^{{0.1177}}\ast {{{\rm{W}}}_{{\rm{t}}}}^{(-{0.1257})}\ast M{{T}_{t-2}}^{{1.7258}}+{0.7675}{P^{\prime} }_{t}$$

### Discussion

**Figure 7:**
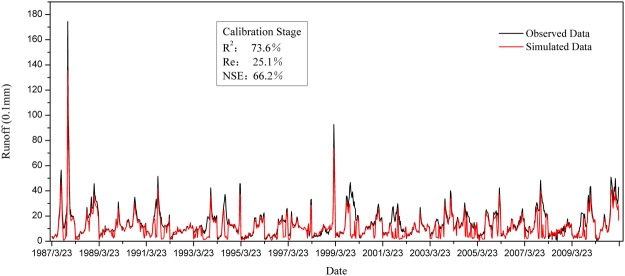
Comparison between observed data and simulated data in calibration stage.

**Figure 8:**
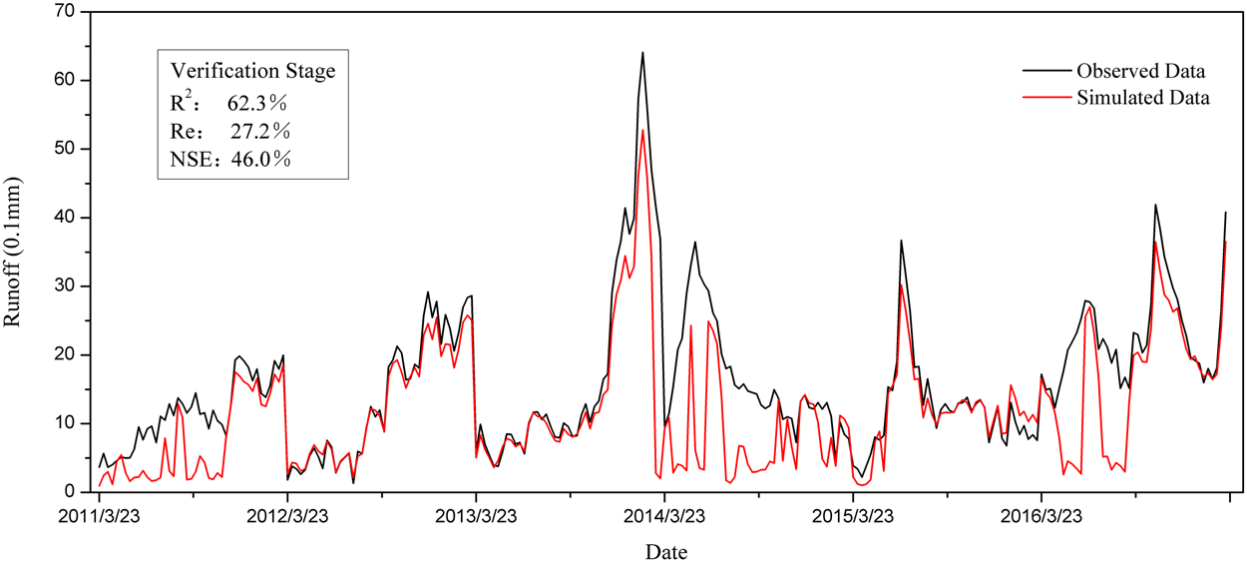
Comparison between observed data and simulated data in verification stage.

Our study area is subject to seasonal frozen soil, where the extreme little snow year was screened through the amount of accumulated snow from November 1 in the previous year to March 31 of the current year based on the meteorological data. We established that March 23 to May 4 is the appropriate period to study snowmelt runoff based on base flow segmentation by the ECK method. The global sensitivity analysis method based on BP neural network is used to identify SR_T0_, W_T0_, P_T0_, and MT_T2_ as the factors that have the greatest effect on snowmelt runoff in the study area. Based on the generalized empirical formula, data from 1987–2010 was taken as the training sample to estimate the empirical formula of snowmelt runoff in the study area with GAS. The simulation results for the calibration period are shown in Fig. 7. The formula performed reasonably well based on R^2^ of 73.6%, Re of 25.10%, and NSE of 66.2% when comparing the observed to the simulated runoff. Data from 2011–2016 were used as a test sample to validate the calibrated empirical formula, with results shown in Fig. 8. Generally, the model showed an acceptable performance with R^2^, Re, and NSE values of 62.3%, 27.2%, and 46.0%, respectively, for the verification period. Therefore, the model has potential value for the estimation of snowmelt runoff into the Baishan reservoir.

Temperature was often used as the dominant parameter for runoff calculation in the snowmelt period during previous analyses of factors affecting snowmelt runoff^21,23^. Because temperature monitoring data are easy to obtain, it is advantageous to use temperature data as an input parameter for a snowmelt model. However, there is a complex correlation between radiation and air temperature, so it is also important to discuss the effect of radiation on runoff during snowmelt period. In this paper, we demonstrated that the snowmelt runoff process is more sensitive to radiation than air temperature, which suggests that radiation cannot be ignored in snowmelt runoff studies. Snowmelt runoff was more sensitive to average temperature than maximum or minimum temperature. The model proposed in this paper exhibited an acceptable performance for both calibration and validation periods. However, there are some differences between the simulated and observed runoff, which may be because of inability to adequately model runoff derived from snow cover or frozen soil. An improved mathematical representation of frozen soil runoff would further improve the accuracy snowmelt runoff estimation for the spring.

## Conclusions

(1) The snowmelt period was identified from March 23 to May 4 through base flow segmentation using the ECK method combined with a series of statistical analyses. (2) By applying a global sensitivity analysis based on the BP neural network, the critical factors affecting snowmelt runoff included daily radiation, daily wind speed, daily mean temperature, and daily precipitation. Radiation is the most essential factor for simulating snowmelt runoff, and because precipitation is the direct source of runoff it must be accurately represented. (3) Time-lag effects on snowmelt runoff varied by factor including daily mean temperature (2-day lag), daily maximum temperature (2-day lag), and daily minimum temperature (1-day lag). (4) The empirical formula can be divided into two sections, the first one reflects the effects of accumulated snow under radiation on runoff, wind and temperature, and the second one reflects the direct effect of precipitation on runoff. (5) Radiation and temperature are directly related to runoff, while we can see that the exponent of wind speed is negative, so wind speed is inversely related to runoff. (6) Output from the current empirical formula has a relative error of <30%. Hence, if high-resolution climatic data are provided, then our empirical formula can give a reliably forecast snowmelt runoff.
